# Suicide among physicians: what do we know about it?

**DOI:** 10.1192/j.eurpsy.2023.2373

**Published:** 2023-07-19

**Authors:** R. P. Vaz, J. Martins, A. L. Costa, J. Brás, R. Sousa, E. Almeida, J. Abreu, N. Castro, R. Andrade, N. Gil

**Affiliations:** 1 Departamento de Psiquiatria e Saúde Mental, Centro Hospitalar Tondela Viseu, Viseu, Portugal

## Abstract

**Introduction:**

The prevalence of mental illness has increased worldwide over the past few years. At the same time, and even in the sense, there is also an increase in suicide rates with special incidence in certain risk groups, among which health professionals stand out.

In this particular group, physicians seem to represent a class particularly vulnerable by the stress and demand associated with it, but also by access and knowledge about potentially lethal means.

For this very part, they have a higher risk of suicide than the general population.

**Objectives:**

This paper aims to better understand the phenomenon of suicide among physicians and identify which medical specialties are most vulnerable.

**Methods:**

Bibliographic research in the Pubmed® database using the terms “suicide and physicians”.

**Results:**

The data obtained from the scientific literature consulted indicate that physicians have a higher risk of suicide than the general population, with greater emphasis on females who have higher rates compared to males.

Work factors that translate into higher levels of demand and stress combined with easy access and knowledge about the use of potentially lethal means seem to contribute very significantly to this phenomenon. Perfectionist personality traits with a high sense of responsibility and duty are also important characteristics that place these professionals in a position of greater vulnerability.

With regard to the different medical specialties, anesthesiology, psychiatry and general and family medicine are the ones with higher suicide rates among the medical class.

**Conclusions:**

The risk of suicide, although admittedly high in the medical class, is not homogeneous among different countries, being naturally influenced by the satisfaction/gratification obtained in the performance of their profession. In this sense, countries such as Switzerland and Canada show higher levels of professional satisfaction. In the opposite direction, dissatisfaction in the exercise of clinical activity is associated with higher levels of fatigue and burnout.

Medical women, due to the need to combine the responsibility of family tasks with professional responsibility, are at greater risk.

In this sense, it is necessary to develop strategies that are more appropriate for the prevention and early identification of suicide risk situations that can be experienced not only by improving working conditions but also by better addressing professionals suffering from mental disorders.

**Disclosure of Interest:**

None Declared

